# Peroxisomal Import Reduces the Proapoptotic Activity of Deubiquitinating Enzyme USP2

**DOI:** 10.1371/journal.pone.0140685

**Published:** 2015-10-20

**Authors:** Katharina Reglinski, Marina Keil, Sabrina Altendorf, Dominic Waithe, Christian Eggeling, Wolfgang Schliebs, Ralf Erdmann

**Affiliations:** 1 Institut für Biochemie und Pathobiochemie, Abteilung Systembiochemie, Ruhr-Universität Bochum, D-44780 Bochum, Germany; 2 Wolfson Imaging Centre, Weatherall Institute of Molecular Medicine, University of Oxford, Headley Way, Oxford, OX3 9DS, United Kingdom; Heinrich-Heine-Universität Düsseldorf, GERMANY

## Abstract

The human deubiquitinating enzyme ubiquitin-specific protease 2 (USP2) regulates multiple cellular pathways, including cell proliferation and apoptosis. As a result of alternative splicing four USP2 isoenzymes are expressed in human cells of which all contain a weak peroxisome targeting signal of type 1 (PTS1) at their C-termini. Here, we systematically analyzed apoptotic effects induced by overexpression and intracellular localization for each isoform. All isoforms exhibit proapoptotic activity and are post-translationally imported into the matrix of peroxisomes in a PEX5-dependent manner. However, a significant fraction of the USP2 pool resides in the cytosol due to a weaker PTS1 and thus low affinity to the PTS receptor PEX5. Blocking of peroxisomal import did not interfere with the proapoptotic activity of USP2, suggesting that the enzyme performs its critical function outside of this compartment. Instead, increase of the efficiency of USP2 import into peroxisomes either by optimization of its peroxisomal targeting signal or by overexpression of the PTS1 receptor did result in a reduction of the apoptotic rate of transfected cells. Our studies suggest that peroxisomal import of USP2 provides additional control over the proapoptotic activity of cytosolic USP2 by spatial separation of the deubiquitinating enzymes from their interaction partners in the cytosol and nucleus.

## Introduction

Dynamic ubiquitination is involved in the regulation of many cellular processes such as cell proliferation, differentiation and apoptosis. Ubiquitin (Ub), a protein of 76 amino acids, is covalently attached to target proteins by E3 Ub-ligases. Poly-ubiquitination leads to degradation of these proteins by the Ubiquitin-Proteasome pathway [1]. Removal of the ubiquitin moiety by specific hydrolyzing enzymes called DUBs (deubiquitinating enzymes) increases the steady-state level of the target protein and thus, stimulates the activity of the cellular pathway in which the ubiquitinated protein is involved. Many USPs have a regulatory function in cell death or growth signaling cascades and are involved in the pathogenesis of human diseases [2].

**Fig 1:**
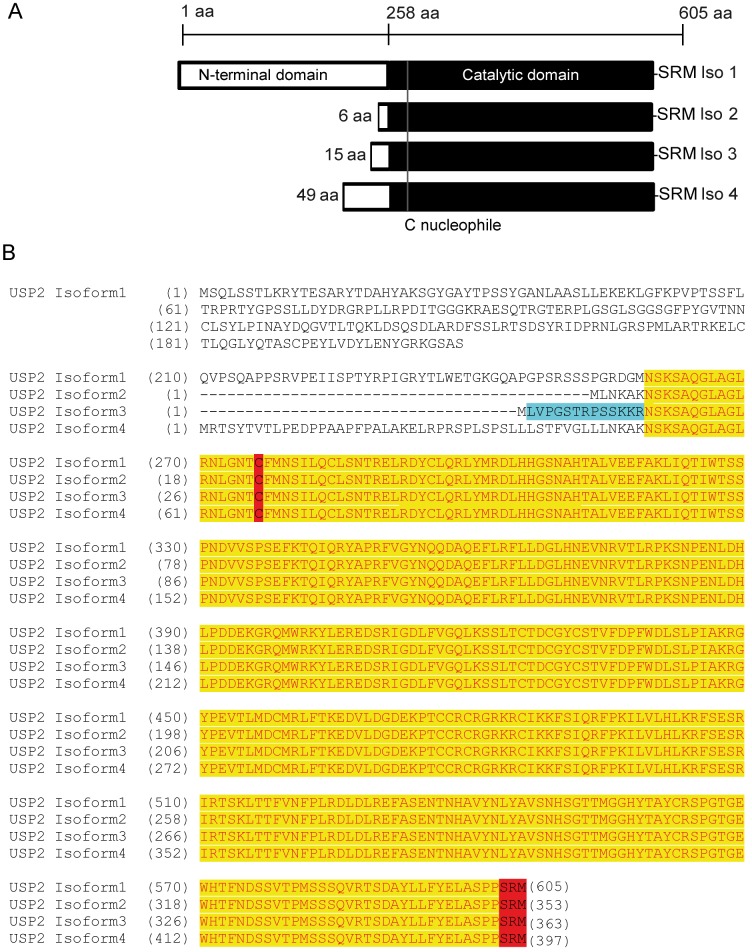
Sequence alignment of the four different isoforms of USP2. A: Schematic overview of four isoforms of human USP2. The sequence of the C-terminal region containing the catalytic domain (in black) and the peroxisomal targeting signal (SRM) is identical for all four isoforms. The isoforms differ in length and composition of the N-terminal domain (in white). B: Sequence alignment of the four USP2 isoforms. During this study, antibodies have been raised against a 13mer peptide representing the N-terminal amino acids of isoform 3 (blue). The catalytic domain is highlighted in yellow. The catalytically critical cysteine residue as well as the C-terminal peroxisomal targeting sequence–SRM are shown in red.

One prominent example is the deubiquitinating enzyme USP2 of which four isoforms were identified (UniProtKB–O75604). All isoforms have an identical C-terminal region, containing the catalytic domain, but differ in length and amino acid composition of the N-terminal regions (**Fig 1)**.

USP2 isoform 1 regulates distinct cellular processes by de-ubiquitination of different substrates, including key targets for tumor suppression such as Mdm2 and MdmX [3, 4]. These proteins are deregulated in many human cancers and exert their oncogenic activity predominantly by inhibiting the p53 tumor suppressor. Other USP2 targets involved in tumorigenesis are Aurora-A [5], Cyclin D1 [6], Cyclin A1 [7] and EGFR (epidermal growth factor receptor) [8]. These findings are in line with the result that USP2-1 is overexpressed in 44% of all prostate carcinomas [9] and contributes to resistance against treatment with Cisplatin in tumor cells [3, 10, 11]. Because of its oncogenic function USP2 has been discussed as a target for anti-cancer therapies [12].

However, the specific roles of different isoforms of USP2 on the regulation of cell homeostasis is controversially discussed. In this respect, isoform specific effects on NF-kB signaling have been reported [13]. USP2-1 and USP-3 mediate TNFα-induced apoptosis by stabilizing members of NFκB signaling pathway, including RIP1 and TRAF2 [14, 15] and TRAF6 [16]. In addition, AIF (apoptosis inducing factor) was also identified as a target for USP2-1[17]. Caspase-dependent apoptosis caused by overexpression of USP2 was first described for USP2-2 [18] but also for isoform 1 [15].

All USP2 isoforms comprise at their C-terminus the tripeptide sequence SRM, which is a potential peroxisomal targeting signal of type 1 (PTS1). These targeting signals are characterized by the C-terminal consensus sequence (S/A/C)(K/H/R)(L/M) [19, 20]. Accordingly, peroxisome association of GFP-tagged USP2 isoforms 1 and 4 has been reported in hepatocytes [21]. USP2 is the only known human peroxisomal protein (as listed in PeroxisomeDB2.0 database [22], which uses the sequence–SRM as PTS1. Interestingly, the specific targeting sequence–SRM can be found at the carboxy terminus of all USP2 homologs in vertebrates, while non-vertebrate homologs carry PTS1-signals with larger sequence variance.

Peroxisomal proteins carrying PTS1 signals are recognised after synthesis and folding by the cytosolic import receptor PTS1 receptor PEX5, which binds PTS-containing proteins in the cytosol and targets them to the peroxisomal membrane. Here PEX5 becomes part of a transient translocation pore [23], enabling the import of folded, even oligomerized proteins. Upon translocation of the cargo protein into the peroxisomal matrix, membrane-integrated PEX5 becomes monoubiquitinated at the cytosolic side of the membrane. This modification is required for the ATP-dependent release of the receptor to the cytosol [24, 25]. At the end of the receptor cycle, the receptor is deubiquitinated, which makes it available for another round of import. This step is catalyzed by the cytosolic deubiquitinating enzyme USP9X, which plays a role in the PEX5 import cycle [26]. Besides USP9X, USP2 is the second DUB with a peroxisomal association. However, the function of this peroxisomal association is still obscure, especially as all known substrates of USP2 are located in the cytosol or nucleus.

Here, we analyzed the function and intracellular localization of USP2. All isoforms can induce apoptosis and are targeted into the matrix of peroxisomes. Inhibition of peroxisomal import did not interfere with the proapoptotic function of USP2, suggesting that USP2 deubiquitinates apoptotic target proteins at other cellular sites, presumably nucleus and cytosol. The proapoptotic activity of USP2 was decreased upon increased targeting of USP2 to peroxisomes. Our studies indicate that apoptosis can be modulated by peroxisomal import of USP2, resulting in its spatial separation from its target proteins in the cytosol and nucleus.

## Results

### All four USP2 isoforms are translocated into peroxisomes

**Fig 2:**
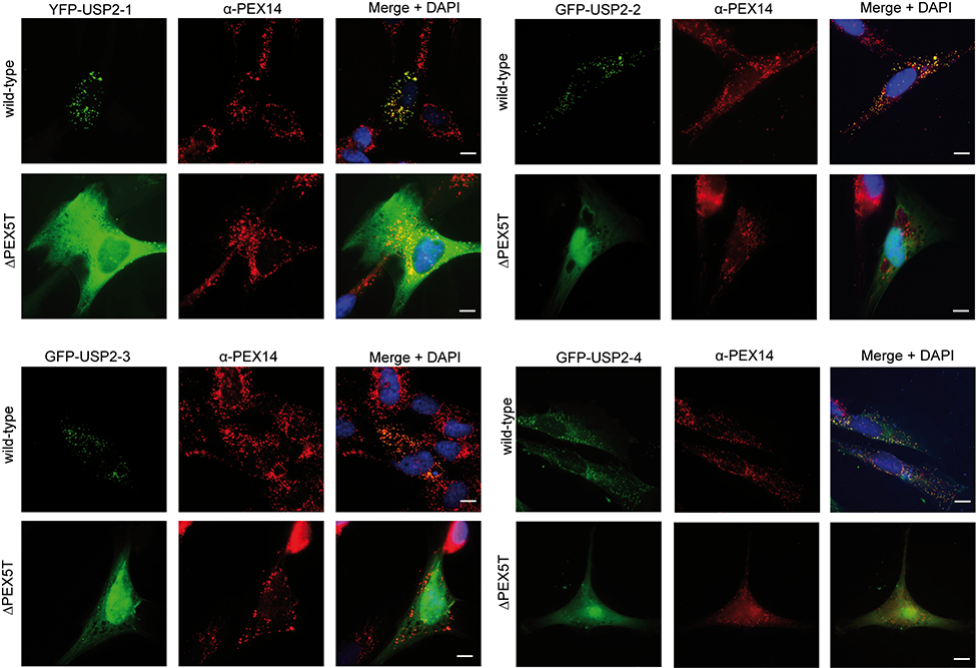
All USP2 isoforms localize to peroxisomes. Fluorescence microscopy images from wild-type fibroblasts and PEX5-deficient fibroblasts, transfected with YFP-USP2-1, GFP-USP2-2, GFP-USP2-3 and GFP-USP2-4 (green). Congruent fluorescent pattern of the labelled USP2 isoforms and PEX14 indicate peroxisomal localization. All four USP2 isoforms are associated with peroxisomes, when expressed in wild-type fibroblasts. The cytosolic and nuclear fluorescent pattern, which is not congruent with the PEX14 staining, indicates a mistargeting as seen in the PEX5 deficient fibroblasts. Cells were counterstained with DAPI to mark the nucleus. Scale bar: 10 μm.

To investigate the localization of USP2 isoforms, each isoform was fused to the C-terminus of fluorescent proteins. These fusion constructs were expressed in normal fibroblasts and PEX5-deficient fibroblasts derived from a Zellweger patient (**Fig 2**). A punctate pattern of GFP-USP2 and colocalization with the peroxisomal marker protein PEX14 was seen upon expression of all four isoforms in normal fibroblasts, indicating that they localize to peroxisomes (Fig 2, wild-type). In contrast, no punctate pattern but a diffuse background staining was observed for all USP2-GFP isoforms in PEX5-deficient cells, indicative of a cytosolic localization and demonstrating that the peroxisomal localization depends on a functional PTS1 receptor (Fig 2, ΔPEX5T). A non-peroxisomal localization in normal fibroblasts was also observed when GFP was fused to the C-terminus of USP2 isoforms. In this case, the C- terminal peroxisomal targeting sequence is blocked by the tag and thus not accessible for the receptor (data not shown).

**Fig 3:**
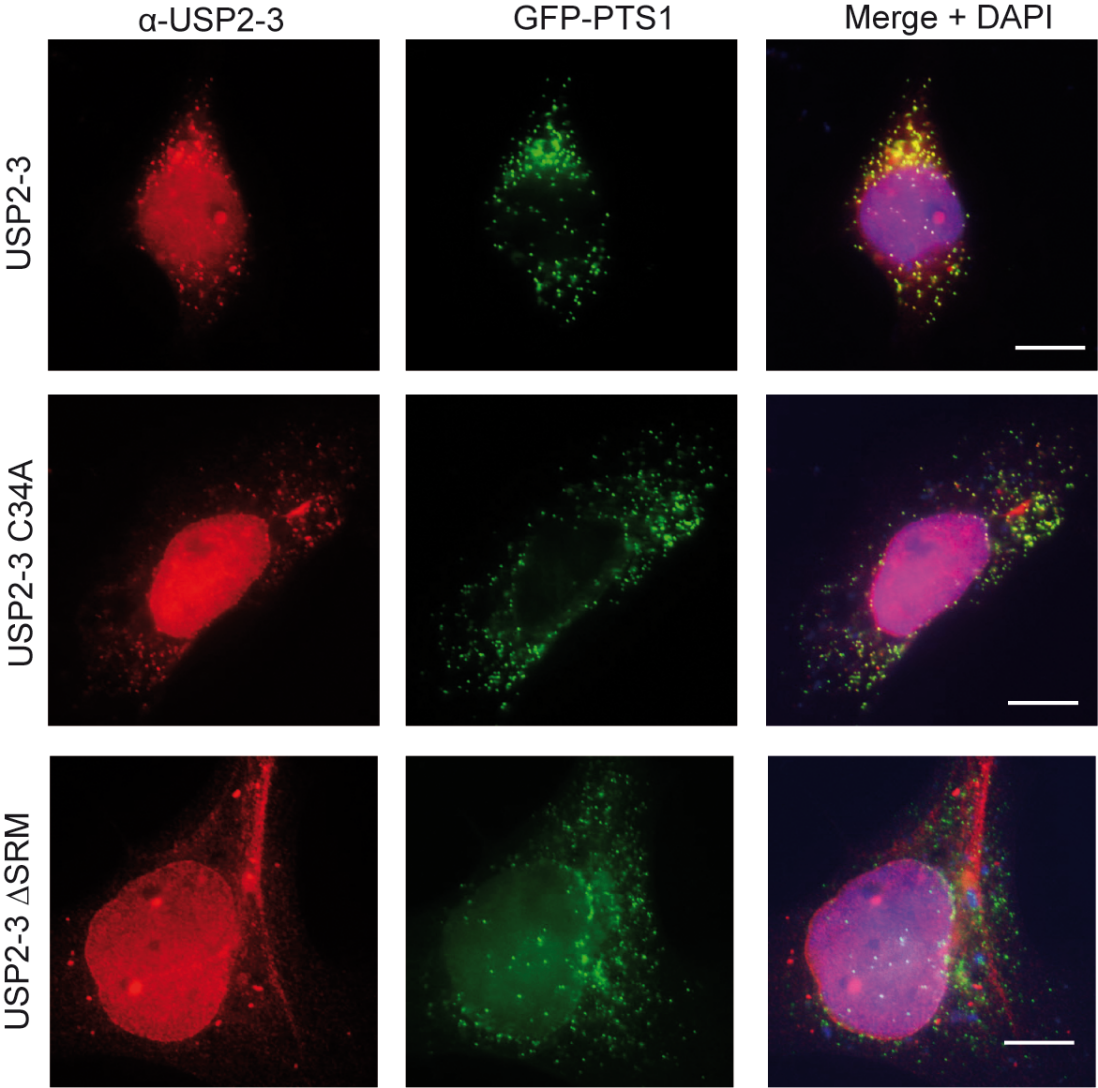
Untagged USP2-3 shows a PTS1-dependent peroxisomal localisation. Immunofluorescence microscopy images of HEK-293 cells, which were co-transfected with the peroxisomal marker protein GFP-PTS1 and either USP2-3, the catalytically inactive variant (USP2-3 C34A), or the PTS1 deficient variant (USP2-3 ∆SRM) as indicated. The overexpressed USP2-3 variants were detected with a peptide antibody raised against the N-terminal isoform-specific region of USP2-3 (red). USP2-3 and the catalytically inactive variant are partially localised to peroxisomes, cytosol and nucleus, while USP2-3 (ΔSRM) is found only in cytosol and nucleus. Peroxisomes are labelled by the GFP-PTS1 (green). Scale bar: 10 μm.

To address the possibility that the N-terminal fusion proteins affect localization, i.e. by masking other targeting signals or binding sites, we raised specific peptide antibodies against USP2 isoform 3 (Fig 1 and **S1 Fig**) and localized the untagged plasmid-encoded protein by immunofluorescence microscopy in HEK-293 cells. The congruent fluorescence pattern of USP2-3 and GFP-PTS1 showed that the USP2 Isoform 3 partially localized to peroxisomes. However, the co-localization of the USP2 isoform 3 with DAPI and the overall background staining indicated that the protein also localized to the nucleus and the cytosol, respectively (**Fig 3**). It looks like that the untagged USP2-3 is localized also in the nucleus (Fig 3), whereas the GFP-tagged protein is not (Fig 2). Therefore, the N-terminal tagging might interfere with the nuclear localization of the protein.

A congruent punctate fluorescence pattern with the peroxisomal marker was also observed for the catalytically inactive USP2 mutant (USP2-3 C34A), indicating that peroxisomal targeting of USP2 is independent of its catalytic function (Fig 3).

In contrast, deletion of the PTS1 signal (USP2-3 ∆SRM) did result in the loss of the peroxisome association (Fig 3). This result shows that the C-terminal tripeptide sequence SRM is essential for peroxisomal targeting of USP2. This result also corroborates the finding that the peroxisomal targeting of USP2 depends on the presence of the PTS1 receptor PEX5 (see above).

**Fig 4:**
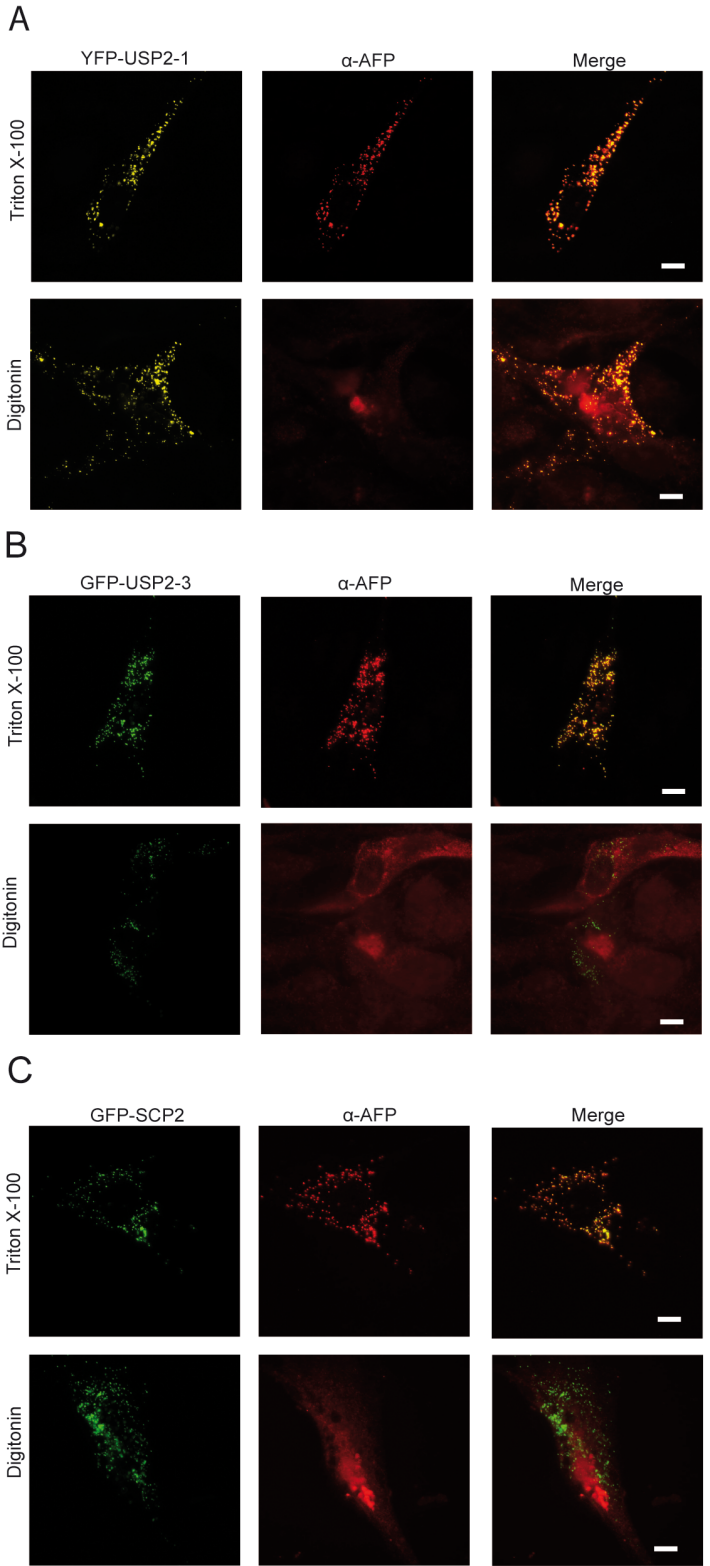
USP2 is a peroxisomal matrix protein. Immunofluorescence microscopy images of wild-type fibroblasts, containing YFP-USP2-1 (**A**), or GFP-USP2-3 (**B**) or GFP-SCP2, a peroxisomal matrix protein (**C**). Immunofluorescence microscopy was performed using antibodies against fluorescent proteins (AFP). Cells were incubated either with 1% Triton X-100, permeabilising all cellular membranes, or Digitonin (25 μg/ml), permeabilizing only the plasma membrane. Using Triton X-100, peroxisomal matrix proteins become accessible for the antibodies, as indicated by the congruent staining of the fluorophore (green) and the AFP antibody (red). Using Digitonin, only proteins facing the cytosolic compartment can interact with the antibodies, leading to a diffuse staining of the AFP antibody, when the fluorescent protein is compartmentalized into cell organelles. YFP-USP2-1 and GFP-USP2-3 could only be detected by the AFP antibody after permeabilization with Triton X-100, indicating that these proteins enter the peroxisomal lumen. Scale bar: 10 μm.

Next, we addressed the question whether USP2 enters the lumen of the peroxisome or remains at the cytosolic surface of the membrane where the PTS receptor becomes ubiquitinated (**Fig 4**). To this end, fibroblasts expressing YFP-tagged USP2 Isoform1 (**Fig 4A**) and or GFP-tagged isoform 3 (**Fig 4B**) or GFP-SCP2 as peroxisomal control (**Fig 4C**) were permeabilized either with 1% Triton X-100, resulting in lysis of all cellular membranes, or 25 μg/ml Digitonin, which only permeabilized the plasma membrane but not membranes of organelles. Upon treatment with Digitonin, exogenously added antibodies have no access to intraorganellar proteins as these are shielded by the membrane. Upon treatment with Triton X-100, the antibodies will have access to all cellular proteins. Without any detergent, the autofluorescence of both isoforms of the tagged USP2 as well as of the GFP-SCP2 revealed a punctate peroxisomal fluorescence pattern. Immunofluorescence microscopy with anti-AFP antibodies that recognize the tagged isoforms 1 and 3 of USP2 as well as the peroxisomal GFP-SCP2 revealed a congruent punctate pattern of autofluorescence and immunofluorescence only in Triton-permeabilized cells but not in Digitonin-permeabilized cells. Accordingly, isoform 1 and isoform 3 of USP2 are not accessible to the exogenously added antibodies upon Digitonin permeabilization, demonstrating that both isoforms reside in the peroxisomal matrix.

### The C-terminal tripeptide of USP2 is not essential for deubiquitination of Ub-PEX5

**Fig 5:**
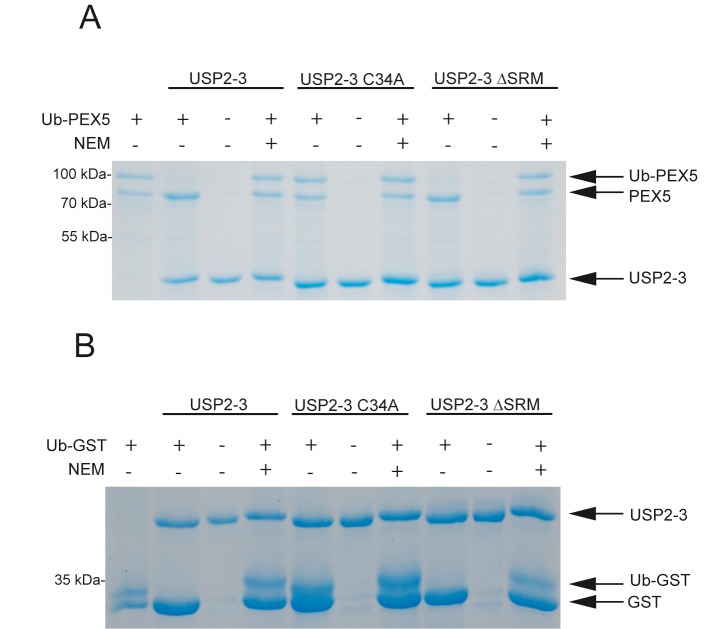
*In vitro* deubiquitination assay. Purified USP2-3, its catalytically inactive variant (C34A) and a variant lacking the PTS-1 (∆SRM) were tested for their ability to deubiquitinate Ub-PEX5 (**A**) and Ub-GST (**B**) *in vitro*. Equal amounts of USP2 and the substrate (200 μg) were incubated for 1h at 37°C followed by SDS-PAGE and Coomassie staining. **A**: A recombinant Ub-PEX5 fusion construct was designed to mimic monoubiquitinated PEX5. Here the first 12 amino acids of PEX5 are replaced by Ubiquitin. PEX5 is monoubiquitinated at the cysteine in position 11. The thioester bond of the original monoubiquitinated PEX5 is replaced by a peptide bond in the Ub-PEX5 model substrate. The recombinant Ub-PEX5 appears as a double band. The upper band of purified Ub-PEX5, represents Ub-PEX5 and the lower one supposedly deubiquitinated PEX5. Ub-PEX5 shifted to the lower mass upon incubation with USP2-3 and USP2-3 ∆SRM, indicative for the deubiquitination of these constructs. This shift was not seen with the catalytic inactive USP2-3 C34A, indicating that this construct was not capable to deubiquitinate Ub-PEX5. The deubiquitination could be inhibited by N-ethylmaleimide (NEM). **B**: Ubiquitin fused to GST was used as a control substrate. The upper band represents the entire fusion construct, while the lower band represents deubiquitinated GST. As Ub-PEX5, also Ub-GST shifted to the lower mass upon incubation with USP2-3 and USP2-3 ∆SRM, indicative for the deubiquitination of these constructs. Again, the catalytic inactive USP2-3 C34A, was not capable to deubiquitinate Ub-GST and the deubiqutination could be inhibited by N-ethylmaleimide (NEM).

In search for peroxisomal USP2-targets for deubiquitination, we focused on the peroxisomal import receptor PEX5, which is transiently ubiquitinated during the receptor cycle. Accordingly, we considered the PTS1 of USP2 to be a specific interacting module to enable efficient removal of mono-Ub from PEX5 during its receptor cycle. To test the influence of the targeting signal on the proteolytic activity, we purified recombinant USP2-3 and a catalytically inactive variant (USP2-3 C34A) as well as PTS1-deleted form (USP2-3 ∆SRM). As model substrate for monoubiquitinated PEX5, a fusion protein consisting of ubiquitin N-terminally fused to amino acids 12 to 639 of PEX5L was designed. Monoubiquitination of PEX5 takes place at a cysteine at position 11. The resulting thioester-bond between ubiquitin and PEX5 is replaced by a corresponding peptide bond in the Ubiquitin-PEX5 fusion protein used here. This fusion protein was purified and tested as substrate for USP2-3 in a deubiquitination assay (**Fig 5**). Recombinant ubiquitinated GST was used as another model substrate. Fig 5 shows that the upper bands, representing either Ub-PEX5 (**Fig 5A**) or Ub-GST (**Fig 5B**), disappear in the presence of USP2. Thus, both substrates are efficiently deubiquitinated by USP2. Deubiquitination was inhibited by mutation of the catalytic cysteine (C34A) of USP2 as well as in the presence of N-ethylmaleimide (NEM), which inhibits cysteine-proteases by modification of the catalytically active cysteine.

Interestingly, Ub-PEX5 is still deubiquitinated by mutant protein USP2-3 ∆SRM. Thus, deletion of the PTS1 of USP2 has no visible effect on the deubiquitination of Ub-PEX5. These results suggest that the interaction between the PTS1 of USP2 and PEX5 is not required for the deubiquitination of PEX5. Ub-GST was also efficiently deubiquitinated by USP2-3 ∆SRM. Taken together the data support the assumption that the PTS1 of USP2 is not required for deubiquitinating activity of USP2 but primarily serves as a targeting signal to direct the USP2 isoforms into the matrix of peroxisomes.

### Replacement of the PTS1-signal from USP2-3 increases its import efficiency

USP2-3 without a fluorescent tag shows a localization distributed between the cytosol, nucleus and a partial peroxisomal fraction (Fig 3). Even upon overexpression, this distribution is not usual for peroxisomal proteins, leading to the assumption that the peroxisomal import of USP2-3 might be inefficient.

**Fig 6:**
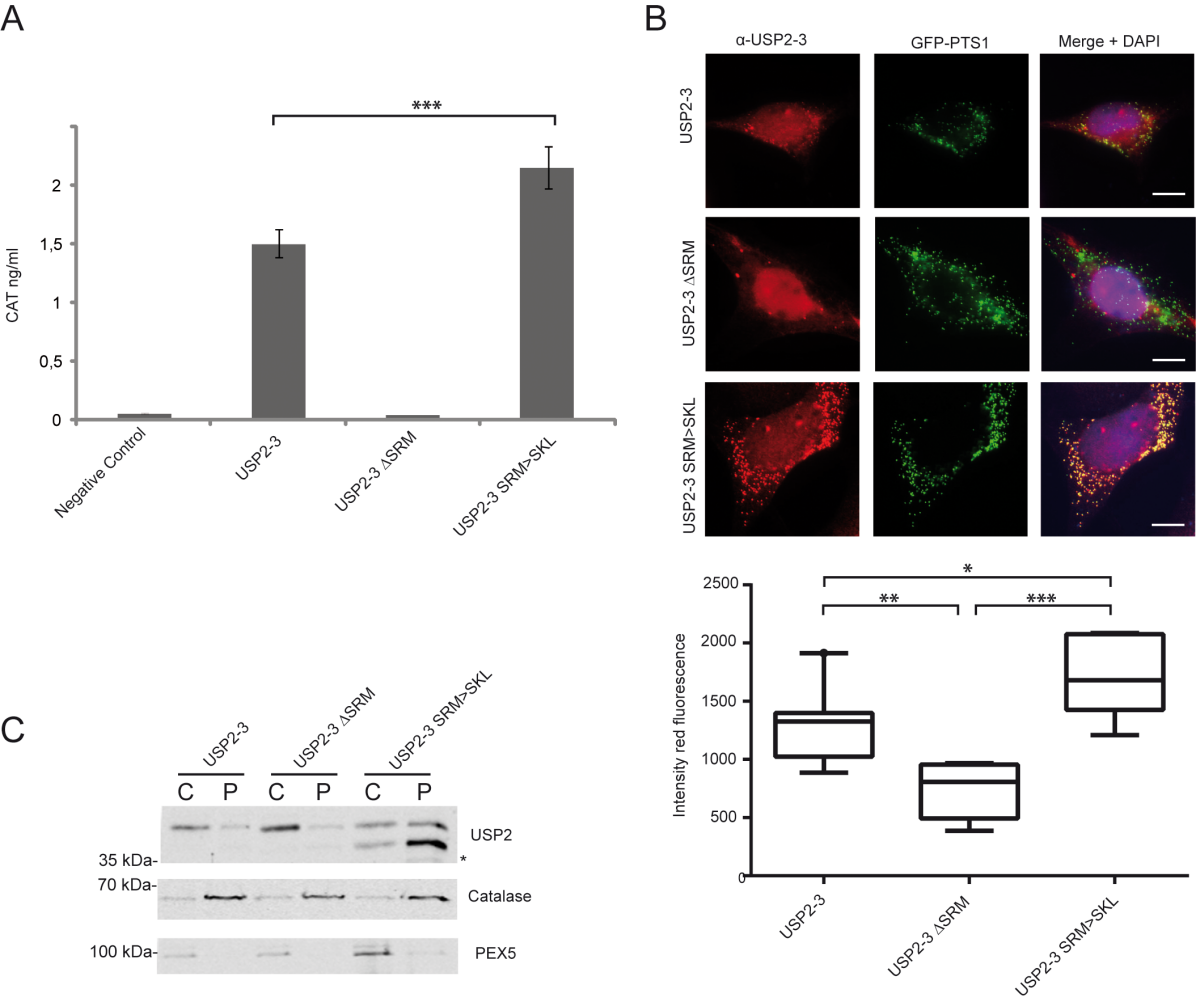
The efficiency of the peroxisomal import of USP2-3 is increased by exchange of its targeting signal. **A**: Mammalian Two-Hybrid analysis of the interaction of PEX5 and USP2-3 variants with different C-terminal sequences. HEK-293 cells were transfected with PEX5, fused to the activation domain, and indicated variants of USP2-3, fused to the binding domain. The interaction between both proteins was monitored by expression of the reporter protein chloramphenicol transferase (CAT). Expression was quantified by a CAT-Elisa assay. The results show that USP2 interacts with PEX5. The PTS1 has a critical role for the PEX5 interaction, as there is no CAT-expression detected when the PTS1-signal is deleted (ΔSRM). In contrast, there is an increased interaction when the PTS1 of USP2 is replaced by the more common PTS1 signal SKL. The statistical significance of the differences between the analyzed USP2-3 variants in their ability to interact with PEX5 was found using a one-way ANOVA test (p<0.0001) and pairwise comparisons performed using the Tukey’s multiple comparison test. The Tukey’s comparison test revealed a significant increase in the expression of the reporter protein after replacement of the PTS1-signal (USP2-3 SRM>SKL) when compared to USP2-3 (***p<0.005). Mean values are shown with standard deviation from three independent experiments which were performed in triplicate for each USP2 variant. **B**: USP2-3 SRM>SKL is predominantly localized in peroxisomes. Immunofluorescence microscopy images of HEK-293 cells, transfected with USP2-3, USP2-3 **Δ**SRM or USP2-3 SRM>SKL as indicated. USP2 variants were visualized with an isoform specific antibody (red). Most of USP2-3 and USP2-3 **Δ**SRM localized to the cytosol and nucleus while the predominant peroxisomal localization of USP2-3 SRM>SKL is indicated by its clear colocalization with the peroxisomal marker GFP-PTS1 (green). Statistical analysis of peroxisomal localization of the different USP2-3 variants by fluorescence intensity measurement in the areas positive for peroxisome staining (bottom panel). For the statistical analysis, nine cells of each condition were analysed. The intensity of the red fluorescence was measured in regions where the green fluorescence was above a threshold (peroxisomes, indicated by the GFP-PTS1 marker protein). The intensity of the red fluorescence in the area of peroxisomes is presented in a boxplot, IQR 25–75 percentile, Whiskas 10–90 percentile. The peroxisomal localization of USP2-3 SRM>SKL, indicated by a higher intensity of the red fluorescence in the region of peroxisomes, is significantly higher than for USP2-3, as indicated by an one way ANOVA analysis and a multiple comparison (Tukey’s) test, (*p<0.05, **p<0.01, ***p<0.005). Scale bar: 10 μm. **C**: Localization of USP2 variants by subcellular fractionation studies. HEK-293 cells, transiently transfected with the indicated USP2-3 variants were subjected to subcellular fractionation studies by differential centrifugation. After permeabilization of the plasma membrane with 25μg/ml Digitonin, the cell organelles were sedimented by centrifugation. The resulting supernatant contained released cytosolic proteins, while permeabilized cells including organelles are present in the sediments. The results show that USP2-3 and USP2-3 ∆SRM are mostly localized in the cytosol (C) with only a small portion associated with the sedimented organelles (P). The exchange of the PTS1 signal results in an increased organelle localization of USP2-3 SRM>SKL, indicated by its predominant presence in the sediment. Catalase was used as peroxisomal marker. Immunodetection was carried out with antibodies against USP2, catalase and PEX5.

The efficiency of peroxisomal import is regulated by the affinity of the PTS1-protein to the import receptor PEX5. By mammalian two-hybrid analysis, a clear binding between PEX5 and USP2 was observed, whereas no interaction was detected when the PTS1-sequence is deleted (ΔSRM) (**Fig 6A**). The two-hybrid signal, measured as expression of a reporter enzyme, was even stronger, when the supposedly weak PTS1 of USP2 (-SRM) was replaced by the strong PTS1 sequence -SKL. The results of the two-hybrid analysis indicate that the interaction between USP2 and PEX5 depends on the quality of the PTS1 and is significantly increased by conversion of the sequence.

The peroxisomal localization of the USP2-3 SRM>SKL construct was verified by immunofluorescence microscopy with USP2-3 specific-antibodies and validated by statistical analysis (**Fig 6B).** Accordingly, USP2-3 SRM>SKL localizes to peroxisomes without the diffuse background staining of cytosol as seen before for USP2-3 containing the weak (–SRM) PTS1. To further quantify the subcellular distribution of USP2 between peroxisomes and the cytosol, we carried out subcellular fractionation studies of HEK-293 cells, which were transiently transfected with USP2-3, USP2-3∆SRM and USP2-SRM>SKL (**Fig 6C**). After incubation with 25 μg/ml Digitonin for permeabilization of the plasma membrane, cytosolic proteins were separated from organelles by centrifugation. In accordance with results of the immunofluorescence microscopy (**Fig 6B**), the USP2-3 variants with and without PTS1 signal, USP2-3 and USP2-3 ∆SRM, were mostly found in the cytosolic fraction. Replacement of the -SRM sequence by -SKL increased the amount of particulate USP2-3 to approximately 50%.

Taken together, these findings indicate that wild-type USP2-3 is mostly located in the cytosol, where it can interact with its various substrates, but a part of the expressed protein is transported into peroxisomes by the PTS1 receptor PEX5. This partial cytosolic localization seems to be due to the weak peroxisomal targeting signal of USP2 as exchange of the weak PTS1 against a strong one significantly increased the peroxisomal import efficiency.

### Peroxisomal targeting influences the proapoptotic function of USP2

**Fig 7:**
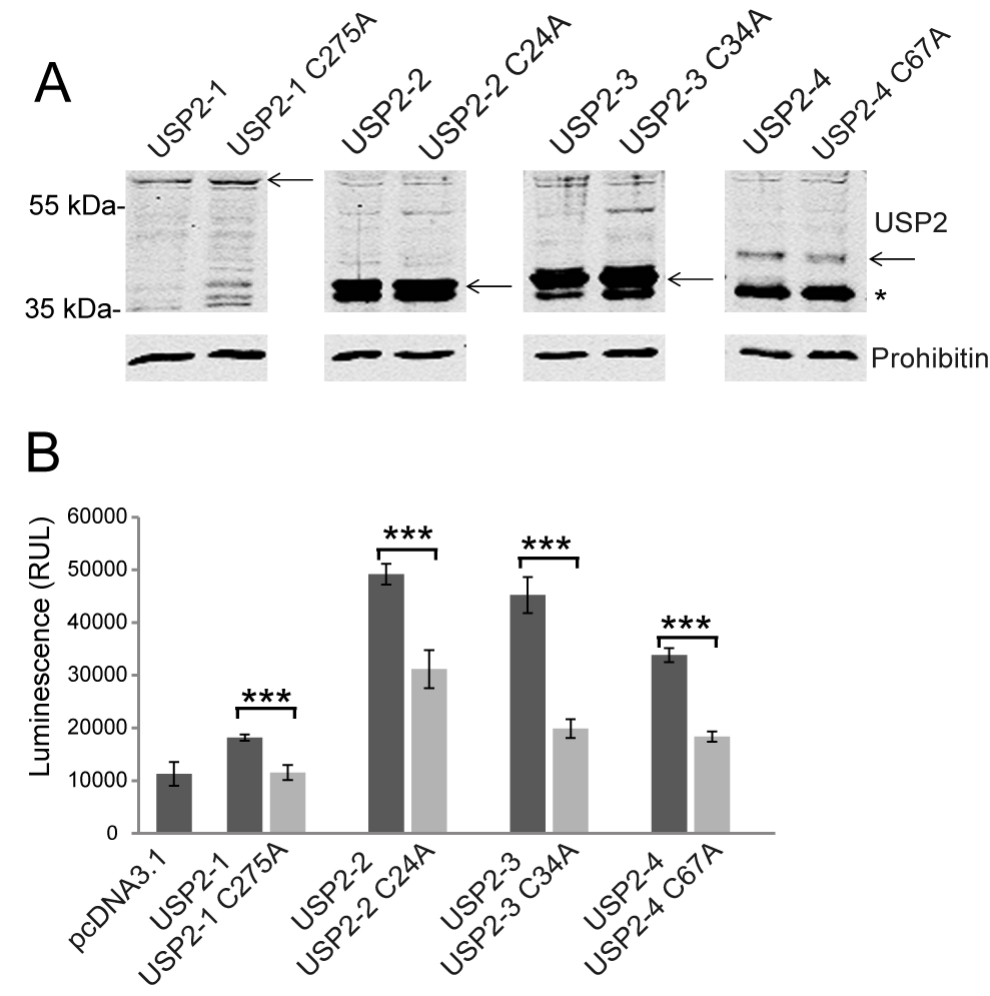
All isoforms of USP2 exhibit proapoptotic activity. **A**: The different isoforms of USP2 and their catalytic mutants were overexpressed in HEK-293 cells. Expression was analyzed by immunoblotting using USP2-X antibodies. The molecular masses of the isoforms are: USP2-1: 68 kDa; USP2-2:40 kDa, USP2-3: 41 kDa; USP2-4: 45 kDa. Each full lengths isoform is denoted by an arrow. The asterisk indicates a degradation product of around 37 kDa, which is common for all isoforms. Prohibitin was used as a loading control. **B**: All Wild-type USP2 isoforms but not their catalytically inactive mutants induce cell death. HEK-293 cells were transfected with the control plasmid (pcDNA3.1) or plasmids expressing the indicated USP2 variants. The induction of apoptosis was monitored by a caspase activity assay, using a luminescent substrate for Caspase 3 and 7. In comparison with the vector control, the caspase activity was significantly increased after expression of each of the USP2 isoforms. Mean values are shown with standard deviation from three independent experiment, which were performed in triplicate for each construct. Two way ANOVA analysis showed that the proapoptotic effect of USP2 depends on the isoform as well as on the activity of the enzyme (p<0.0001). (RUL = *Relative Light Unit*).

One function assigned to isoform 2 is its proapoptotic activity induced by overexpression [18]. In order to gain more insight into apoptotic function of all four USP2 isoforms, we overexpressed each of them in HEK-293 cells and monitored the induction of apoptosis using a caspase 3/7 assay. As negative control, the same expression plasmids harboring substitutions of the active site cysteine (Fig 1, C nucleophile), were transfected. Immunoblot analyses revealed that the USP2 isoforms and their catalytically inactive variants are expressed at comparable levels (**Fig 7A**). Our results indicate that all USP2 isoforms exhibit proapoptotic activity. Cells expressing isoform 3 exhibited an 2.2 fold increase in caspase activity in comparison to the inactive control (USP2-3 34A), whereas the other isoforms show an increase of 1.6 to 1.8 fold increase in caspase activity in comparison to their inactive controls.(**Fig 7B**).

**Fig 8:**
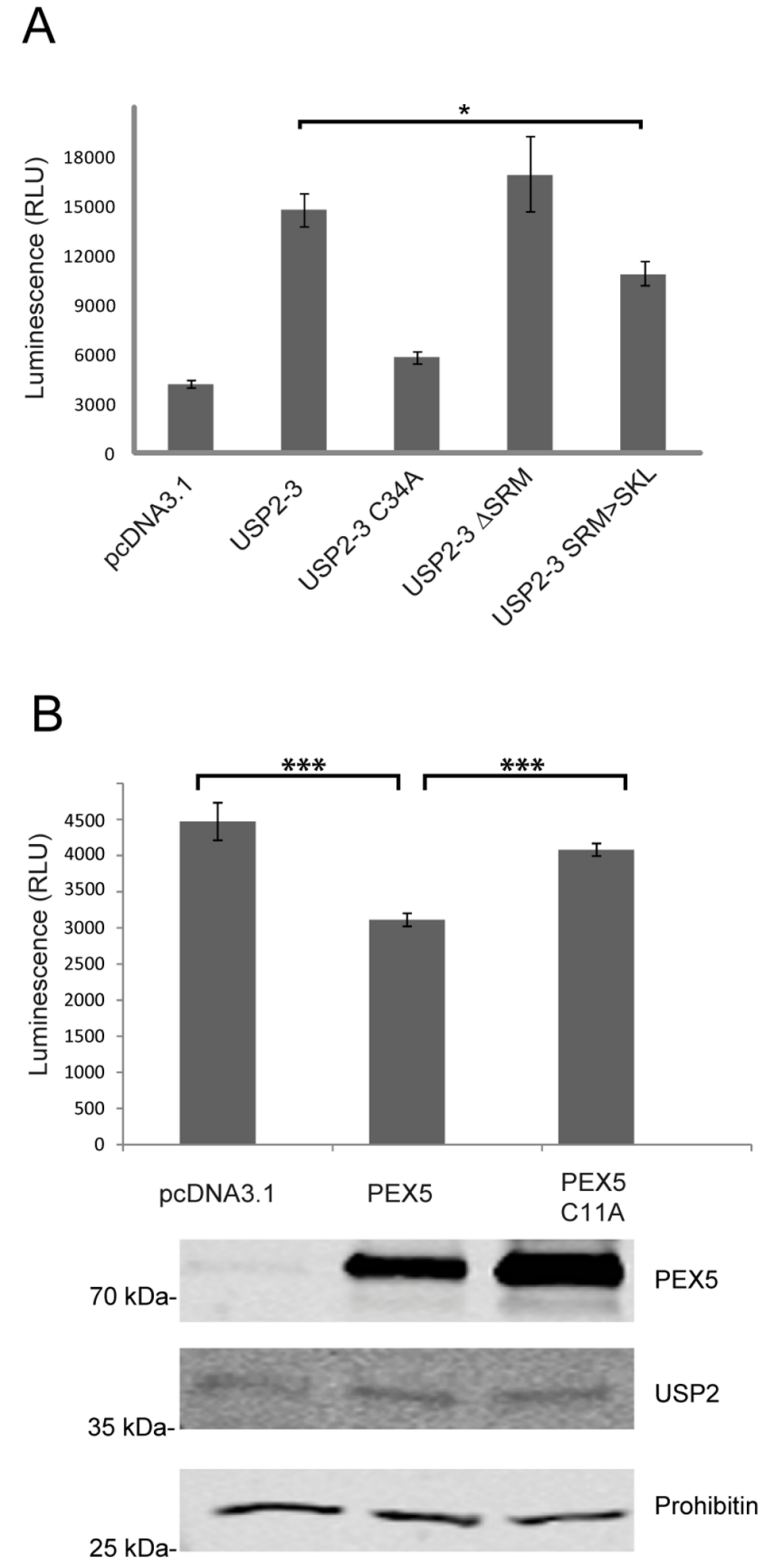
The proapoptotic effect of USP2-3 overexpression is reduced by optimization of the peroxisomal import. **A**: Activity of caspase 3/7 in HEK-293 cells after expression of different USP2-3 variants. Cells were transfected with the empty control plasmid pcDNA3.1 or plasmids, encoding the indicated USP2-3 variants. 24 h after transfection caspase activity was measured by a luminescence assay. Expression of USP2-3 did result in an increase of the caspase activity, while caspase acitivity upon expression of the inactive USP2-3 C34A variant was only slightly above the control. The apoptotic effect of the different USP2-3 variants differ significantly, which was shown by one way ANOVA analysis (p<0.0001). However, in comparison to the unmodified protein, the caspase activity was significantly decreased upon expression USP2-3 SRM>SKL, which was shown by a Tukey’s multiple comparisons test (*p<0.05). Mean values are shown with standard deviation from three independent experiments, which were performed in triplicate for each USP2 variant. **B**: Activity of Caspase 3/7 of FlpUSP2-3 cells after transfection with the empty vector or plasmids coding for PEX5 or mutated PEX5 C11A, which does not support peroxisomal protein import. 48 h after transfection, caspase activity was monitored by a luminescence assay (upper panel). The caspase activity significantly differs upon the expressed PEX5 variants (one way ANOVA analysis; p<0.001). Tukey’s multiple comparisons test revealed that in comparison to the control (empty vector), transfection with the import defective PEX5 C11A mutant had no significant effect on the caspase activity, but was significantly decreased after transfection of wild-type PEX5 (***p<0.005). Shown are mean values with standard deviation from three independent experiments, which were performed in triplicate for each construct. Expression of the different PEX5 variants was analyzed by immunoblotting (lower panel). Prohibitin was used as a loading control. PEX5, USP2-3 and Prohibitin were detected with specific antibodies. (RUL = *Relative Light Unit*).

To study the effect of peroxisomal deposition on proapoptotic activity of USP2, we carried out a caspase activity assay with USP2-3 and its variants with modified PTS1 signals (**Fig 8A**). To this end, USP2-3 variants lacking the PTS1 signal (USP2-3 ΔSRM) or containing the optimized PTS1 (USP2-3 SRM>SKL) were expressed in HEK-293 cells. Expression of USP2-3 resulted in a two-fold increase of caspase-activity, while the catalytically inactive mutant, USP2-3 C34A, did not alter the apoptotic rate (Fig 8A). Deletion of the PTS1 signal, which is essential for peroxisomal localization, (Fig 3, Fig 6) had no significant effect on the apoptotic effect of the non-modified USP2-3. However, when the weak PTS1 was replaced by the optimized PTS1, which targets USP2-3 efficiently to peroxisomes (**Fig 6**), the proapoptotic effect was clearly reduced (Fig 8A). Our data suggest that USP2-3 fulfills its apoptotic activity by interaction with cytosolic and nuclear interacting partners and that this interaction is reduced after peroxisomal import.

To test the hypothesis, we expressed the PTS1 receptor PEX5 in a cell line stably expressing USP2-3 at a high level (FlpUSP2-3) and monitored their apoptotic activity. Cells overexpressing PEX5 did exhibit lower caspase activity than non-transfected cells (**Fig 8B**), most probably due to the increase of the protein import rate. As negative control, we also expressed an inactive PEX5 variant, PEX5L C11A which possesses an intact PTS1-binding site but recycling of the receptor from the peroxisomal membrane is impaired, leading to a complete loss of PTS1-dependent protein import [27]. In contrast to wild-type PEX5, expression of the inactive PEX5 mutant did not affect the apoptotic rate of these cells (Fig **8**B).

Altogether, the data suggest a correlation between the proapoptotic activity of USP2-3 and efficiency of peroxisomal import.

## Discussion

Here we show that all four isoforms of the ubiquitin-specific protease USP2 are imported into peroxisomes in a PEX5-dependent manner. However, peroxisomal import of USP2 is not very efficient so that a significant portion remains in the cytosol or is transported in the nucleus. It is not known whether USP2 performs a special function in peroxisomes, especially as there is no evidence for the presence of ubiquitinated proteins inside peroxisomes. However, the receptor cycle of PEX5 requires ubiquitination and de-ubiquitination, which takes place at the outer surface of peroxisomes or in the cytosol. The cytosolic deubiquitinating enzyme USP9X has been found to catalyze the deubiquitination of PEX5 [26]. However, it is not known whether also other deubiquitinating enzymes can perform this function for PEX5. Taking into account that USP2 interacts with the import receptor PEX5, deubiquitination of monoubiquitinated PEX5 could have been a peroxisome-related function of the enzyme. However, our *in vitro* analyses suggest that the presence of PTS1 has no effect on the selectivity of USP2, which does not support this hypothesis.

The peroxisomal localization of USP2 raises the question, whether there are other peroxisomal substrates or intraperoxisomal signaling pathways, which involve ubiquitination and deubiquitination. From studies in plants, it has been suggested that during the transition from seed glyoxysomes into leaf peroxisomes, the matrix enzymes isocitrate lyase and malate synthase are extracted from peroxisomes in an ubiquitin-dependent manner [28–30]. In the yeast *Hansenula polymorpha* as well as in human cells, it has been shown that ubiquitination of membrane proteins, in particular PEX3 triggers autophagic degradation of peroxisomes [31–34]. In the light of the high variety of ubiquitin-conjugated processes at the outer surface of peroxisomes, we cannot exclude that USP2 modulates these processes. However, the intraperoxisomal localization of this enzyme does not coincide with such a function.

The lack of intraperoxisomal targets for USP2 led to the consideration that the peroxisomal import could serve as a regulating step for cytosolic activities of USP2. In this case, deubiquitinating enzymes could be separated from their cytoplasmic substrates in a fast and efficient manner resulting in faster degradation of target proteins. Here we could show that higher apoptotic rates upon their overproduction is a common feature of all USP2 isoforms. Since the endogenous isoforms are expressed at a lower level, it remains unclear whether the observed proapoptotic activity upon overexpression reflects their physiological function. However, our data suggest that the proapoptotic activity of the enzymes correlates with their subcellular localization. Increasing the efficiency of peroxisomal import, either by overexpression of the PTS1 receptor or by exchanging the weaker PTS1 of USP2 by a stronger one, reduced the apoptotic rate of cells overexpressing USP2. In accordance with the cytosolic localization of known USP2 target proteins involved in apoptosis, it can be speculated that the decreased apoptotic cell rate is a result of the shortened time in which USP2 stays in the cytosol. However, increased efficiency of peroxisomal import could not completely abolish the proapoptotic activity of the plasmid-encoded USP2. In this respect, it seems noteworthy that PTS1 proteins are not immediately imported into peroxisomes upon their synthesis and folding but can stay in the cytosol for minutes before they end up in peroxisomes [35–39]. Since receptor binding via PTS1 does not abolish enzymatic activity of cargo enzymes [40], it seems possible that USP2 can interact with cytosolic factors before the import process into peroxisomes is finished.

In this context, it is noteworthy that endogenous USP2 is targeted to peroxisomes by a unique PTS1 sequence -SRM, which shows a weaker interaction with the cycling PTS1 import receptor PEX5, in comparison to the common PTS1 signal -SKL (Fig 6A). PTS1 signals with a low affinity to PEX5 often exhibit dual steady-state localization in the cytosol and peroxisomes, as demonstrated for alanine-glyoxylate aminotransferase (AGT) [41], human and yeast catalases [42–44], or plant glutathione reductase [45]. Very recently, stress-induced peroxisomal targeting has been shown for a plant MAP kinase phosphatase with the non-canonical PTS1 sequence–SAL [46].

There are also examples for peroxisomal proteins of which the efficiency of targeting signals is affected by posttranslational modifications. A peroxisomal protein that performs a critical function in the cytosol is the osmolyte-generating glycerol 3-phosphate dehydrogenase (Gpd1) of baker´s yeast. Gpd1 carries a PTS which is regulated by phosphorylation [47]. For human epoxide hydrolase, it has been demonstrated that dimerization prevents peroxisomal import, most likely by masking the PTS1 signal [48]. Mutations of epoxide hydrolase, which interfere with its dimerization, show an increased peroxisomal localisation. These mutations are found in patients with familiar hypercholesterolemia and insulin resistance in diabetes type II. Therefore, epoxide hydrolase is an enzyme whose peroxisomal import is reinforced by a disease-related mutation. Here the misfolded protein might be removed from the cytosol by peroxisomal import to prevent harm for the cell, tissue or organism.

Accordingly, we hypothesize that peroxisomes might function as a metabolic valve for proapoptotic USP2. On the one hand, import of USP2 into peroxisomes seems to be an efficient way for its depletion from the cytosol and thus modulation of cell fate. This way is probably much faster and less energy dependent than the degradation of USP2 by the proteasome, as the peroxisomal proteins can be imported in folded, and even oligomerized manner with no need for energy- and time-consuming unfolding. On the other hand, a defect of peroxisomal protein import or even release of USP2 from defective peroxisomes because of cell injury or dysfunction would increase the cytosolic pool of USP2, which might contribute to the initiation of apoptosis.

## Methods

### Plasmids and cloning strategies

*Escherichia coli* strain TOP10 (Invitrogen, Carlsbad, USA) was used for DNA cloning. The different USP2 isoforms and variants where cloned into the pcDNA3.1 (Invitrogen, Carlsbad, USA) vector. USP2-1 was amplified from HsCD00044595 (PlasmID, Harvard Medical School) with the oligonucleotides RE3543 and RE3793 and subcloned into NotI/HindIII digested pcDNA3.1. USP2-2 was amplified from USP2-3 in pcDNA 3.1 with oligonucleotides RE4090 and RE3793 and cloned into pcDNA3.1, using HindIII and NotI restriction sites. USP2-3 was amplified from cDNA USP2 in pBluescript (RZPD, Berlin IMAGp998C111690Q1) with primer pair RE3181/RE3173 and subcloned into XhoI and ApaI digested pcDNA3.1. The substitution of the catalytic cysteine in all four isoforms has been carried out by site-directed mutagenesis, using primers RE2670 and RE2671. The modification of the PTS1 signal of USP2-3 was carried out by amplification of USP2-3 with modified reverse primers, RE3804 in case of USP2-3 ΔSRM and RE4273 in case of USP2-3 SRM>SKL.

USP2-3 was subcloned into the pPROEX expression vector for the heterologous expression in *E*. *coli* BL21 (DE3) by amplification with the oligonucleotides RE2156 and RE2157 and use of the restriction sites SpeI and XhoI. The variants of USP2-3 for the preparation of the recombinant proteins USP2-3 C34A and USP2-3 ΔSRM were created by site-directed mutagenesis with the primer pairs RE2670/RE2671 (USP2-3 C34A) and RE3077/RE3078 (USP2-3 ΔSRM), respectively.

For the localisation studies, the USP2 isoforms 2, 3 and 4 were subcloned into pEGFP-C1 (Clonetech, Mountain View, USA) using primers RE4269 and RE4274 and restriction sites HindIII/SalI (USP2-2) and RE2019/RE2020 with restriction sites XhoI and NotI (USP2-3). In case of USP2-4, the DNA-fragment encoding the N-terminal 49 amino acids of USP2-4 were synthesised (GenSkript, USA) and cut with HindIII and EcoRI. This fragment was ligated into the HindIII/EcoRI-digested GFP-USP2-3 vector, resulting in a GFP-USP2-4 construct. YFP-USP2-1 has been constructed by a Gateway reaction between USP2-1 in Gateway Entry Vector pDONR221 and destination-vector Vivid Colors™ pcDNA™6.2 N-YFP-DEST (Invitrogen, Carlsbad, USA). The peroxisomal marker GFP-SCP2 has been described elsewhere [49].

The C11A substitution was introduced into the PEX5 sequence by site-directed mutagenesis of pIRES2-PEX5L-eGFP-SKL [50] using oligonucleotides RE4367 and RE4368. The bicistronic vector allowed the validation of peroxisomal import in transfected cells by peroxisomal localisation of the marker Protein GFP-PTS1. For all experiments, the long splice variant of PEX5 (PEX5L) was used. The sequences of all primers are listed in Table 1.

**Table 1:** Sequence of oligonucleotides.

Name	Sequence 5‘ → 3‘
RE2019	TACACCTCGAGCTCTTGTGCCCGGTTCGACTC
RE2020	CATCCGCGGCCGCCTACATTCGGGAGGGCGG
RE3077	CCAGCCCGCCCTAACGCATGTAGCTCGAGGCATG
RE3078	CTCGAGCTACATGCGTTAGGGCGGGCTGGCCAG
RE2156	GACACTAGTTCCCAGCTCTCCTCCACC
RE2157	TATCTCGAGCTACATTCGGGAGGGCGG
RE3543	GATCAAGCTTAATGTCCCAGCTCTCCTCCAC
RE3793	GATCGCGGCCGCCTACATTCGGGAGGGCGG
RE2670	CTTCGAAACCTTGGGAACACGGCATTCATGAACTCAATTCTGCAG
RE2671	CTGCAGAATTGAGTTCATGAATGCCGTGTTCCCAAGGTTTCGAAG
RE3181	ATGCTCGAGATGCTTGTGCCCGGTTCG
RE3173	ATGGGGCCCCTACATTCGGGAGGGCGG
RE3804	GATCGGGCCCTTAGGGCGGGCTGGCCAG
RE4090	GATCAAGCTTATGCTCAACAAAGCCAAGAATTCTAAGAGTGCCCAGG
RE4273	GATCGCGGCCGCCTACAGTTTGGAGGGCGGGCTGGCC
RE4274	GATCGTCGACCTACATTCGGGAGGGCGGG
RE4269	GATCAAGCTTCAATGCTCAACAAAGCCAAGAATTC
RE4367	GATCAAGCTTCTACAGTTTGGAGGGCGGG
RE4368	GCACCCCCGGCTTCGGCCTCCACCAGCT
RE2534	GTCACCATGGAGATCTTCGTCAAG
RE2535	ACTTGCGGCCGCGGATCCACCACCTCTTAGTCTTAAG
RE2536	ATGGTAGGTCTCGGATCCGGGGGTGCCAACCCGCTC
KU1651	CTGCGGCGGCTCACTGGGGCAGGCCAAACAT
RE3803	GATCGCGGCCGCAATGGCAATGCGGGAGCTGG
RE3802	GATCCCGCGGTCACTGGGGCAGGCCAAAC
RE3542	GATCAAGCTTAATGCTTGTGCCCGGTTCGAC
RE3536	GATCAAGCTTCTACATTCGGGAGGGCGGG
RE3763	FATCAAGCTTTTAGGGCGGGCTGGCCAG
RE4330	GATCAAGCTTCTACAGTTTGGAGGGCGGG

### Fluorescence microscopy

Human fibroblast and HEK-293 cells were cultured at 37°C in Dulbecco's modified Eagle's medium supplemented with 10% fetal calf serum, 2 mM L-glutamine, 100,000 U/l penicillin, and 100 mg/l streptomycin at 8% CO2.

Primary PEX5-deficient Zellweger patient cells that are homozygous for c.826C>T (R276T) were kindly donated by R. Wanders (AMC, Amsterdam) and SV40-transfected for immortalization to generate a PEX5∆T cell line [51]. The cells were grown for one day on cover slides in 60 mm tissue-culture dishes and were transfected with expression plasmids using X-Treme Gene HP DNA Transfection Reagent (Roche Diagnostics, Mannheim, Germany). 48 h after transfection, the cells were fixed with 3% formaldehyde in phosphate-buffered saline (PBS), permeabilized with 1% Triton X-100, or 25 μg/ml Digitonin, in PBS, and subjected to immunofluorescence microscopy. Polyclonal rabbit antibodies against PEX14 [52] were used to stain peroxisomes and mouse AFP antibodies (MP Biomedicals, Heidelberg, Germany) to stain fluorescent proteins. Secondary antibodies were conjugated with Alexa Fluor 594 (Invitrogen, Germany). All micrographs were recorded on a Zeiss Axioplan 2 microscope with a Zeiss Plan-Apochromat 63×/1.4 oil objective and an Axiocam MR digital camera and were processed with AxioVision 4.2 software (Zeiss, Jena, Germany). Each experiment was performed at least twice and for each transfection, more than one hundred USP2-expressing cells were inspected for peroxisome morphology and localization of peroxisomal proteins. Representative cells are shown in the figures.

For the statistical analysis of colocalization, an intensity comparison was performed. The intensity measurement was calculated using a custom macro written in Fiji/ImageJ analysis software. A region of interest was drawn around each cell and then an automated threshold (Max Entropy) applied to the green and red channel in this region representing the GFP fluorescence and red labeled USP2-3 antibody, respectively. The location of the pixels above the threshold in the green and red channel are then compared. The sum of the red intensity signal in the pixels above the threshold in the green channel was measured. This gave a measure of overall colocalisation in terms of expression intensity.

### Protein expression and *in vitro* deubiquitination assay

*Escherichia coli* strain BL21 (DE3) was used for protein expression. USP2 Isoform 3 DNA and the variations USP2-3 C34A and USP2-3 ∆SRM were cloned into the pPROEX vector, which allowed fusion to an N-terminal poly-histidine tag.

The Ubiquitin-PEX5 fusion construct consists of *Sc*Ubiquitin with an optimized codon usage [53] and amino acids 13–639 of the human PEX5L, the long splice variant of human PEX5, separated by a Gly-Ser (-GS) linker. His-TEV-ScUb-GS was amplified from YEP96 with the primer pair RE2534 and RE2535 and subcloned into the expression vector pET-24d (modified by G. Stier, EMBL) with the restriction sites NcoI/NotI. *Hs*PEX5 13–639 was amplified from pGD106 [54] with the oligonucleotides RE2536 and KU1651 and ligated into the BamHI/NotI digested His-TEV-ScUb-GS in pET-24d.

BL21 (DE3) cells, transformed with USP2-3 constructs or Ub-PEX5 were grown in LB medium and protein expression was induced by addition of 0.4 mM IPTG. Protein expression was carried out at 30°C for 4 h. Sedimented cells were suspended in buffer A (20 mM potassium phosphate pH 7.4, 20 mM imidazol, 300 mM NaCl and 1 mM DTT) and lysed by sonication. The resulting homogenate was centrifuged (rotor SS34, 45 min, 37.500xg, 4°C) and the supernatant was loaded onto NiNTA column and eluted with an imidazole gradient from 20–1000 mM in buffer B (20 mM potassium phosphate pH 7.4, 300 mM NaCl and 1 mM DTT). The buffers were supplemented with 0.5% Tween 40 in case of the USP2-3 variants.

The Ub-GST construct was bound to GSH-sepharose after cell disruption in buffer (150 mM NaCl; 25 mM potassium phosphate pH 7.5) and sedimentation of cell fragments by centrifugation. The protein was eluted by a buffer, containing reduced glutathione (150 mM NaCl; 25 mM potassium phosphate pH 7.5; 50 mM reduced glutathione).

For the *in vitro* deubiquitination assay, 200 μg of purified USP2-3, USP2-3 C34A or USP2-3 ∆SRM were mixed with 200 μg Ub-PEX5 or Ub-GST in deubiquitination buffer (50 mM TrisHCl pH 7.4; 50 mM MgCl_2_, 1 mM DTT). To inhibit deubiquitination, N-ethylmaleimide NEM was used in a final concentration of 20 mM. The samples were adjusted to a volume of 50μl and incubated at 37°C for 1h. The reaction was stopped by the addition of SDS-sample buffer and the reactions were analyzed by SDS-PAGE followed by Coomassie staining.

### Mammalian two-hybrid assay

The mammalian Matchmaker two-hybrid assay kit (Clontech) was used to generate the desired plasmid constructs. The human PEX5 gene was amplified by PCR with oligonucleotides RE3803 and RE3802 and pGD106 [54] as template. The resulting PCR-fragment was subcloned in the NotI/SacII- restricted pVP16 expression Vector, containing the VP16-activation domain. USP2 Isoform 3 and its variants were cloned in the pM vector, carrying the GAL4-binding domain. USP2-3 was amplified with the oligonucleotides RE3541 and RE3536 and subcloned into SmaI/HindIII digested pM. USP2-3 ΔSRM and USP2-3 SRM>SKL were amplified with a different reverse primers, RE3763 and RE4330, respectively and also cloned into pM via SmaI/HindIII.

HEK-293 cells were cultured and transfected as described above. For the mammalian two-hybrid assay, all three plasmids, pM-USP2-3 variant (0.9 μg), pVP16-PEX5 (0.9 μg), and pG5CAT reporter vector (0.2 μg) were co-transfected. The cells were allowed to grow at 37°C in 8% CO2 for 48 h. After transfection, secreted chloramphenicol transferase (CAT) was monitored using the CAT ELISA kit (Roche Diagnostics, Mannheim, Germany).

### Immunoblot analysis and antibodies

For immunoblot analysis, HEK-293 and FlpUSP2-3 cells were transfected with mammalian expression vectors using the X-TremeGene transfection reagent according to the manufactures instructions. Cell lysates were harvested 24 or 48 h after transfection. Cells were lysed using Reporter Lysis Buffer (Promega) supplemented with protease-inhibitors (Roche Diagnostics, Mannheim, Germany). Lysates were subjected to SDS-PAGE and immunoblot analysis.

Peptide antibodies were raised in rabbits against USP2 by Pineda Antibody Service Berlin, Germany. The peptide antibody which specifically detects isoform 3 (USP2-3 antibody) was raised against the first 13 amino acids of USP2-3, which are unique to this isoform (LVPGSTRPSSKKR) (Fig 1B). Isoform 3 was chosen for a detailed analysis in this study because of its strong proapoptotic effect and the possibility to raise an isoform specific antibody against its N-terminus. Another peptide antibody was raised in rabbits against a peptide from the catalytic domain of USP2-3 with the sequence RDYCLQRLYMRDLHHGSNAH, resulting in antibodies detecting all USP2 isoforms (USP2-X). Polyclonal antibodies against human PEX5 [40] and Prohibitin (Abcam, Cambridge, Great Britain) were used. Fluorescent secondary antibodies were detected by the Odyssey Infrared Imaging System, LI-COR, Lincoln, NE, USA.

### Caspase 3/7 assay

5000 HEK-293 cells were seated in a 96-well plate. After 24h, the cells were transfected with 0.2 μg DNA as described above. The measurement of the caspase activity was carried out 24h or 48h after transfection using the Caspase-Glo 3/7 Assay (Promega), according to the instructions of the manufacturer.

### Miscellaneous

Subcellular fractionation was performed as described elsewhere [55]. The generation of a stable expression cell line expressing USP2-3 was performed according to Bharti *et al*. [51].

### Statistical analysis

Results were analyzed using the Student’s t test, if two groups were compared. Analysis of variances (ANOVA) for a single factor (one-way) was used. Differences between the analyzed grouped were specified by a Tukey’s multiple comparisons test with adjusted p values. All data are expressed as a mean values with standard deviation (+/- SD) value of p < 0.05 was considered significant.

## Supporting Information

S1 Fig Characterization of peptide-derived antibodies against USP2 isoform 3. (PDF)

## Abbreviations

AFP: All fluorescent proteins
DUB: deubiquitinating enzyme
PTS: peroxisomal targeting signal
Ub: Ubiquitin
USP: ubiquitin-specific protease
