# Supplementary material for: Peroxisomal Import Reduces the Proapoptotic Activity of Deubiquitinating Enzyme USP2
Source: PLoS One. 2015 Oct 20;10(10):e0140685. doi: 10.1371/journal.pone.0140685 (PMC4617714; doi:10.1371/journal.pone.0140685)
Supplement: S1 Fig — (PDF) [file pone.0140685.s001.pdf]

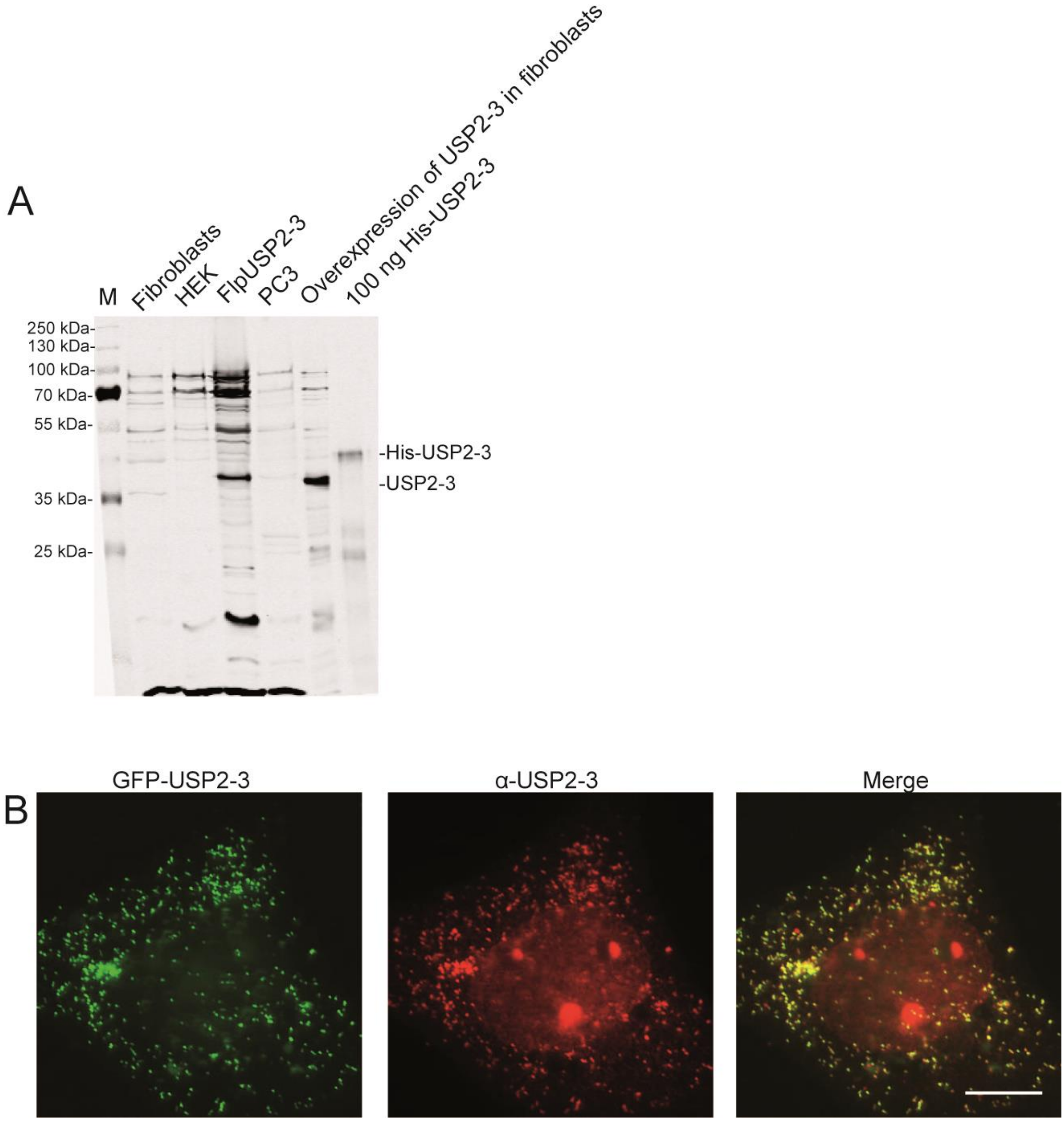

Supporting Figure S1: Characterization of peptide-derived antibodies against USP2 isoform 3.

A: Analysis of the specificity of the antibody against USP2 Isoform 3 by immunoblot analysis. Cell lysates of human fibroblasts, HEK cells, the USP2-3 expressing cell-line (FlpUSP2-3) and the prostate cancer cell line PC3 were tested for the presence of USP2. 100 ng purified His-USP2-3 was analyzed as positive control. USP2 was detected in the positive control, the USP2-3 expressing cell-line (FlpUSP2-3) as well as in human fibroblasts, expressing plasmid-encoded USP2-3. M, molecular mass marker. **B**: Detection of USP2 by fluorescence microscopy. HEK cells were transfected with GFP-USP2-3 expression plasmid and the protein was detected by GFP autofluorescence (green) and immunofluorescence microscopy with the USP2-3 antibodies (red). The merge of the images revealed a congruent punctate fluorescence pattern (yellow). Scale bar: 10 µm.
